# Phospholipases A_1_

**DOI:** 10.3390/ijms12010588

**Published:** 2011-01-18

**Authors:** Gregory S. Richmond, Terry K. Smith

**Affiliations:** 1 Agilent Technologies, Molecular Separations, Santa Clara, CA 95051, USA; E-Mail: gregoryr9@gmail.com; 2 Centre for Biomolecular Sciences, The North Haugh, The University, St. Andrews, KY16 9ST, Scotland, UK

**Keywords:** phospholipase A_1_, phospholipid, lysophospholipid

## Abstract

Phospholipase A_1_ (PLA_1_) is an enzyme that hydrolyzes phospholipids and produces 2-acyl-lysophospholipids and fatty acids. This lipolytic activity is conserved in a wide range of organisms but is carried out by a diverse set of PLA_1_ enzymes. Where their function is known, PLA_1_s have been shown to act as digestive enzymes, possess central roles in membrane maintenance and remodeling, or regulate important cellular mechanisms by the production of various lysophospholipid mediators, such as lysophosphatidylserine and lysophosphatidic acid, which in turn have multiple biological functions.

## 1. Introduction

**Figure 1:**
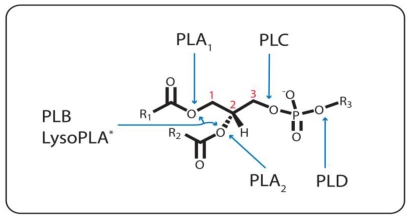
Ester Bond Specificity of the Phospholipases. PLA_1_, PLA_2_, and PLC catalyze the hydrolysis of the ester bond emanating from the *sn*-1(1), *sn*-2 (2), and *sn*-3 (3) carbon, respectively. PLD hydrolyzes the other phosphodiester bond. PLB cleaves both the *sn*-1 and *sn*-2 ester bonds. * = LysoPLA can either be specific for the *sn*-1 or *sn*-2 bond, or both, when one or the other acyl chain is missing. R_1_ and R_2_, (CH_2_)*_n_*CH_3_; R_3_, various headgroups.

Phospholipases form a diverse class of enzymes optimized to hydrolyze phospholipid (PL) substrates at specific ester bonds. Phospholipases vary considerably in structure and function, and as such they are assembled as a group solely on the basis that they are lipolytic enzymes involved in PL metabolism. Two general sets of phospholipases exist, the acyl hydrolases and the phosphodiesterases; and the enzymes within each set are classified according to the cleavage of the ester bond for which they are specific (Figure 1). Phospholipase A_1_ (PLA_1_), phospholipase A_2_ (PLA_2_), phospholipase B (PLB), and lysophospholipase A_1/2_ (LysoPLA_1/2_) constitute the acyl hydrolases, whereas the phosphodiesterases are represented by phospholipase C (PLC) and phospholipase D (PLD).

Phospholipases exist in almost every type of cell analyzed for their presence and most cells contain a multitude of them. For a given PL ester bond, there can be a variety of subtypes of a phospholipase that are specific for it that can either exist as secreted, membrane associated, or in cytoplasmic form. They may also require cofactors for activity, depending on the isoforms. The functions of phospholipases, where known, are as varied as their cellular and tissue localization and properties. Nonetheless, three general functions can be ascribed to the physiologic relevance of phospholipases: (1) they can serve as digestive enzymes, e.g., PLA are ubiquitous in snake and vespid venoms; (2) they can play an important role in membrane maintenance and remodeling, *i.e.*, fatty acid (FA) chains of PLs can be cleaved and exchanged by an acyl hydrolase and an acyltransferase, respectively; and (3) they can regulate important cellular mechanisms, e.g., creation of bioactive lipid molecules used in signal transduction. These three areas of function can be a rather simplistic view accepting that, for example, maintaining acyl composition of membranous PLs can be considered quite an important regulatory function for the cell, even though the reason that cellular membranes require a heterogeneous mixture of PL fatty acids is still not fully understood [1]. These three themes will be explored in more detail in subsequent sections.

Among the different sorts of phospholipases, the most studied and well understood are PLA_2_, PLC, and PLD, all of which play proven important roles in the creation of bioactive lipid molecules. Although this review will focus on PLA_1_, a brief overview of the other phospholipases and their biological significance will also be given to gain perspective.

### 1.1. Phospholipase A_1_

**Figure 2:**
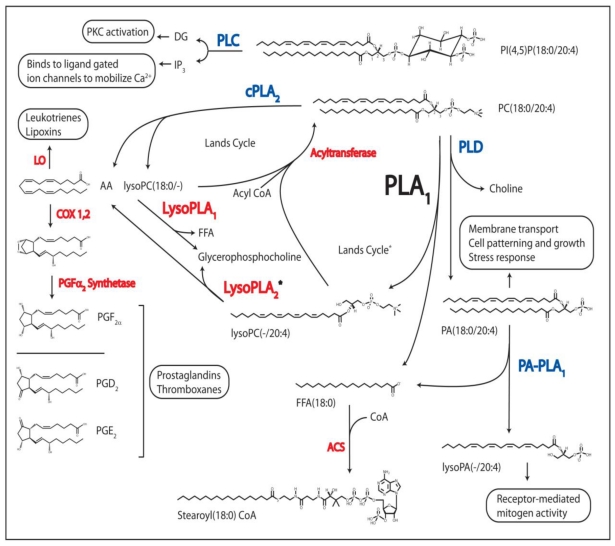
Regulatory Processes Linked to PL Metabolism. The phospholipases known to produce bioactive lipid molecules are shown in blue, and their second messenger metabolites, or their signal-transduced responses, are boxed. Other enzymes utilized in PL and FA metabolism are in red. DAG, diacylglycerol; IP_3_, inositol(1,4,5)phosphate; PKC, protein kinase C; AA, arachidonic acid; LO, lipoxygenase; COX 1,2, cyclooxygenase 1 and 2; PG, prostaglandin; FFA, free fatty acid; CoA, coenzyme A; PLC, phospholipase C; cPLA_2_, cytoplasmic phospholipase A_2_, PLD, phospholipase D; PA-PLA_1_, phosphatidic acid phospholipase A_1_; PLA_1_, phospholipase A_1_, LysoPLA_1_, lysophospholipase A; ACS, acyl-CoA synthetase; PA, glycerophosphatidic acid; PC, phosphatidylcholine; PI, phosphatidylinositol. * = theoretical pathway based on indirect *in vitro* evidence.

PLA_1_ (EC 3.1.1.32) catalyzes the hydrolysis of FAs exclusively at the *sn*-1 position of PLs. A free fatty acid (FFA) and a lysophospholipid (lysoPL) are the products of a PLA_1_ reaction (Figure 2). This class of phospholipase is not well understood, and no crystal structures exist for any true PLA_1_ [2,3]. The assignment of a function for any PLA_1_ from any organism has yet to be firmly established. Historically, the biological role of this acyl hydrolase was often defined by its anticipated participation in the Lands Cycle, which is a deacylation-reacylation cycle that PLs are suspected to undergo in order to remodel their acyl chains to preserve a homeostatic molecular species composition of PLs in membrane bi-layers [4]. However, only one PLA_1_ has ever been directly implicated in a Lands Cycle [5], though this cycle has repeatedly been shown to occur at the *sn*-2 position via the action of PLA_2_, and studies continue to observe the phenomenon [6–10]. The assignment of a similar function for PLA_1_, though potentially valid, cannot be decuded based on experimental data. Understanding of these enzymes is limited, though some progress has been made over the past 20 years [11].

What is intriguing is the observation that despite a theoretical role in the Lands Cycle, none of the PLA_1_ cloned and characterized have been linked to membrane turnover and remodeling roles. On the contrary, there is some evidence to suggest that some PLA_1_ enzymes are virulence factors [12] or act to generate bioactive lysoPL molecules. For example, lysoPL mediators such as lysophosphatidic acid (lysoPA) [13] are second messenger signaling components in pathways that vary in complexity and evolutionary conservation, and they have been implicated in numerous processes such as proliferation, protein transport, differentiation, invasion, and morphogenesis [14]. By activating specific G-protein coupled lysoPL receptors, they are now viewed as key factors in cell-to-cell communication [15]. The synthesis and regulation of the formation of lysoPLs are not fully understood, a reason why PLA_1_ is an important enzyme to study as it could be involved in their formation.

The FA product from a PLA_1_ reaction also has bioactive potential. This has been shown in plants, where a PLA_1_ regulates jasmonic acid biosynthesis. Also, it has been postulated for decades that *sn*-2 arachidonic acid (AA) cleavage from PLs may sometimes be mediated by concerted sequential PLA_1_/LysoPLA_2_ activities [16–24]. No cloned PLA_1_ has been implicated in this alternative route, and therefore it remains circumstantial with regards to the *in vitro* studies, which provide the most indirect evidence [16,22,23].

### 1.2. The Other Acyl Hydrolases: Phospholipase A_2_, B, and Lysophospholipase

PLA_2_ (EC 3.1.1.4) mediates acyl ester hydrolysis at the *sn*-2 position of PLs. PLA_2_ are quite well conserved across *taxa,* and they consist of a broad range of enzymes that segregate into one of eleven groups within the superfamily [25]. Numerous PLA_2_ can contribute to lysoPL signaling events [26] and can even down-regulate the bioactive lysoPL platelet activating factor (PAF) with the *sn*-2 acetate-specific cleavage of PAF by PAF acetyl hydrolase (PAF-AH) [27]. However, PLA_2_ is most noted for its role in initiating the AA cascade (Figure 2) [28]. The *sn*-2 reacylation step of the Lands Cycle appears to be quite specific for AA in a number of cells [29]. Once liberated by a cytoplasmic PLA_2_, AA is converted into over 150 known eicosanoids, including prostaglandins, leukotrienes, and thromboxanes, all powerful local hormones that act as mediators in many important processes such as inflammation in the higher eukaryotes [30]. Numerous crystal structures exist in this class of enzymes whose active mechanism either utilizes a catalytic histidine in a so-called dyad or a catalytic serine in either a dyad or a triad [25,27,31].

PLB is able to hydrolyze both the *sn*-1 and *sn*-2 FAs of PLs (Figure 1). Glycosylation is a common feature of PLBs, such as the one from *Penicillium notatum*, which contains numerous asparagines-linked carbohydrates and possess phospholipase and subsequent lysophospholipase activity [32]. The distinction between PLB and LysoPLA can, rightly so, often be muddled since most PLBs possess lysophospholipase activity [33]. On the other hand, some PLB enzymes have been erroneously annotated. For example, a case in which a purified hamster heart cytosol enzyme clearly displayed both *sn*-1 and *sn*-2 hydrolysis has been referred to as a PLA [34]. Also, the first so-called PLA_1_ to be purified, cloned, and crystallized was the outer-membrane phospholipase A (OMPLA) from *E. coli* and other bacteria [35–37], but it has been known for a long time that it can cleave at both positions *sn*-1 and *sn*-2 of diacyl- or lysoPLs and thus should be considered a phospholipase B [2,24].

LysoPLA (EC 3.1.1.5) detoxifies detergent-like lysoPL intermediates in PL metabolism by removing the remaining acyl chain from the lysoPL (Figure 1). This class of enzymes is also not well characterized. LysoPLA could also be important in the regulation of the amount of bioactive lysoPLs used in receptor-mediated or other signaling mechanisms [38]. Some LysoPLA can function equally well as either a LysoPLA_1_ or a LysoPLA_2_ [17]. Other LysoPLA can deacylate specific species of lysoPL, like those with AA esterfied to the *sn*-2 position, for example [18,39]. Such *in vitro* specificity has helped to fuel the theory that the AA cascade could be initiated by the combined actions of a PLA_1_ and a LysoPLA_2_.

### 1.3. The Phosphodiesterases: Phospholipase C and D

PLC or PI-PLC (EC 3.1.4.11) is mostly known for catalyzing the cleavage of the phosphorylated membrane lipid PI to produce, for example, the intracellular second messengers *sn*-1,2-diacylglycerol [23] and inositol (1,4,5)-trisphosphate (Figure 2). The phosphoinositides are a critically important class of PLs which have been extensively researched, along with the mechanisms by which they are metabolized by PLC [40]. PLC-mediated phosphoinositide production is a key regulating component of ion channels and transporters, and controllers of vesicular trafficking and the transport of lipids between intracellular membranes [41]. Other types of PLC include a secreted form in bacteria that prefers PC (E.C. 3.1.4.3), and a GPI-PLC form found in various organisms that specifically recognises non-inositol-acylated glycosylphosphatidylinositol anchors.

PLD (EC 3.1.4.4) hydrolyzes the *sn*-3 phosphodiester bond of mostly PC to generate a choline molecule and PA. PLD has also been studied in detail due to its link with the production of PA, an intracellular lipid messenger implicated in almost every conceivable aspect of intracellular membrane transport [42]. PLD is also a potential regulator of lyso-PA formation, whose biosynthetic formation is unclear, but could involve both PLD and PLA_1_ [43]. The mode of action and structural characteristics of the various isoforms of PLD are well characterized [42,44].

## 2. Classification of Phospholipase A_1_

Over one hundred years ago accumulation of FFAs was observed upon incubation of pancreatic juice with phosphatidylcholine (PC), and the existence of enzymes responsible for the release of these acids was accordingly proposed. Snake venoms were also found to possess PL-hydrolyzing enzymes when in 1903 cobra venom was found to alter PC into products, which could cause red blood cell hemolysis. These products were later determined to be a PC molecule that was missing a FA, and they were thus termed lysophosphatidylcholine (lyso-PC). Only in the early 1960’s however was the positional specificity of FA cleavage of snake venom determined to be the *sn*-2 position of the glycerol backbone of PLs [44]. Early on, therefore, the study of PLA activity focused on PLA_2_ activity, as it has remained to this day. Nevertheless, phospholipases of type A were linked to those enzymes cleaving FAs from PLs, and the designation A_1_ and A_2_ was employed to signify the acyl hydrolysis from either the *sn*-1 or *sn*-2 position of the glycerol moiety, respectively [45]. It wasn’t long before intracellular PLA activities were beginning to be discovered in numerous organisms and tissues, thus opening the way for the possibility that these enzymes were more than just digestive catalysts.

PLA_1_ specifically hydrolyzes *sn*-1 acyl esters from PLs producing FFAs and lysoPLs (Figure 2). However, many PLA_1_ enzymes exhibit some, usually much lower, LysoPLA activity and neutral lipase activity (*i.e.*, hydrolyzing diacylglycerol or triacylglycerol), yet still preferring the *sn*-1 cleavage site. Enzymes having high PLA_1_ relative to LysoPLA and neutral lipase activity are thus considered true PLA_1_ enzymes, and they can only be designated as such by empirical determination after some level of purification or, more preferably, by cloning and characterization of the recombinant enzyme. The vast amount of results from early studies involving the detection of PLA activity from crude tissue or subcellular fractions were flawed due to the inability to precisely determine what enzyme activities are present and how to differentiate particular enzyme activities. Competing and/or downstream enzyme activities on lipid substrates can mask the activity of the enzyme being tested.

PLA_1_ enzymes have not been formally classified into groups, as have the other major phospholipases. The major obstacle is the lack of available sequence due to only a relatively small proportion of PLA_1_ enzymes having been cloned thus far (Table 1). The only invariable feature of PLA_1_ seems to be a lipase consensus motif in the peptide sequence, which follows the amino acid residue pattern [LIV]-{KG}-[LIVFY]-[LIVMST]-G-[HYWV]-S-{YAG}-G-[GSTAC], inside of which resides a common GXSXG motif, containing the catalytic serine of the active site, as part of a catalytic triad.

PLA_1_ has been considered as a descendent of neutral lipases, and several PLA_1_ sequences show substantial sequence similarity to the well characterized pancreatic, hepatic, and endothelial lipases [43,46,47]. Other PLA_1_ sequences show no similarity to lipase sequences beyond their lipase consensus pattern, nor do they show similarity to other PLA_1_ sequences. Furthermore, eukaryotic lipases possess two domains whereas prokaryotic lipases only contain one, in which there is little if any conservation in sequence (compare alignments in [48–51].

## 3. Mammalian Phospholipase A_1_

A large number of crude protein preparations from mammalian cell and tissue homogenates were enriched for PLA_1_ activity in the 1970’s and 1980’s, but many of these reports describe PLA_1_ activity of uncertain origin. A good example of this is the characterization of a “phospholipase A_1_” from the plasma of rat liver that was found to hydrolyze triacylglycerols, diacylglycerols, and monoacylglycerols, in addition to PLs [71]. This “phospholipase A_1_” was located on the surface of endothelial cells and was shown to play a role in high density lipoprotein metabolism, and two papers were published characterizing its “phospholipase A_1_” activity. Interestingly, relatively recently a new member of the lipase family was cloned from endothelial cells and a recombinant form was shown to possess PLA_1_ activity [72]. Endothelial lipase most likely accounts for the majority of the PLA_1_ and lipase activities previously reported from enriched rat liver endothelial cell fractions.

It us now generally accepted that mammals have six extracellular and three intracellular PLA_1_ enzymes [50]. The extracellular PLA_1_s consist of phosphatidylserine (PS)-specific PLA_1_ (PS-PLA_1_), membrane-associated phosphatidic acid (PA)-selective PLA_1_s (mPA-PLA_1_α and mPA-PLA_1_β), these PLA_1_s may have physiological roles as they produce the lysophospholipids, lyso-PS and lyso-PA known to be lipid mediators with multiple biological functions. The other three extracellular enzymes are either involved in high-density-lipoprotein catabolism of triacylglycerol, hepatic lipase (HL) and endothelial lipase (EL), or digestion of dietary lipids, pancreatic lipase-related protein (PLRP)-2. These three enzymes belong to the lipase gene family showing triacylglycerol-hydrolyzing activity as well as PLA_1_ activity. The intracellular PLA_1_s, KIAA0725p and p125, are conserved in a wide range of organisms and have been implicated in vesicular transport.

### 3.1. Bovine Brain PLA_1_

One of the first phospholipases of the type A_1_ to be purified by column chromatography was from bovine brain [21]. The enzyme was isolated from the soluble fraction and eluted at a molecular mass of 365 kDa from a Sephacryl S-300HR column. Lipases are known for their interfacial activation properties, and the 365 kDa molecular weight obtained could reflect the enzyme’s molecular weight upon association with buffer detergent micelles, or it could represent the enzyme in tetramer form. Bovine brain PLA_1_ migrated as two bands of 112 kDa and 95 kDa by SDS-PAGE. The purified enzyme was shown to be resistant to metal chelators, PMSF, and DFP, but it was inactivated by ZnCl_2_ and enhanced by Ca^2+^, Mg^2+^ and Sr^2+^. The enzyme exhibited broad substrate specificity in mixed micelles made with CHAPS, but the highest specific activity, 23.8 μmol/min/mg, was exhibited against PE(16:0/20:4), and LysoPLA_1_ activity was also observed. A subsequent study showed that PC and PI could also be catalyzed at a high rate by bovine brain PLA_1_ but only in the presence of other PLs like PS, PE, and PA [73]. This same group reported the first chromatographic purification of a LysoPLA_2_ that showed selectivity towards deacylating arachidonate from lyso-PC(−/20:4) [39]. In addition to its LysoPLA_2_ activity, this enzyme also showed PLA_2_ activity, but at a slower rate. Bovine brain LysoPLA_2_ was isolated from the soluble fraction and is an approximately 95 kDa polypeptide. Moreover, it became clear that prior *sn*-1 deacylation of PC(16:0/24:0) by bovine brain PLA_1_ greatly increased the rate of arachidonate deacylation by brain LysoPLA_2_. These results suggested that in bovine brain AA might possibly be generated by sequential PLA_1_/LysoPLA_2_ action, yet this has never been shown *in vivo*. Even more, the physiological substrates for these two enzymes have never been determined so it is impossible to know whether arachidonoyl-substituted PC or PE are in fact hydrolyzed by these enzymes. Unfortunately, not knowing the *in vivo* physiological substrates is a recurring theme in PLA_1_ studies. It is also important to note that the genes encoding these two PLA activities have not been determined.

### 3.2. Bovine Testis PA-PLA

The phosphatidic acid-preferring phospholipase A_1_ (PA-PLA_1_) from bovine testis is well studied. The initial identification of PA-PLA_1_ was from Mono Q fractions of high-speed supernatants from bovine testis, and to a lesser extent in bovine brain [53]. The enzyme displayed preference for PA in TX-100 detergent mixed micelles, and it also displayed a relatively small amount of LysoPLA_1_ activity. At the time of its identification, PA was just beginning to be recognized as a second messenger that could affect a number of cellular processes. The production and, particularly, the attenuation of signaling PA can be mediated by multiple phospholipases that regulate the timing, location, amount, and various molecular species of PA [74]. A 14,000-fold purification of native PA-PLA_1_ was achieved and evidence was provided that the enzyme was a homotetramer of 110 kDa subunits [75]. When the enzyme was examined under the same conditions as the bovine brain PLA_1_ previously reported (*i.e.*, 3–5 mM MgCl_2_) [21], the results showed that PE(16:0/24:0) became the preferred substrate. When MgCl_2_ was removed, PA-PLA_1_ preference for GPA was restored. It is thus possible that the bovine testis PA-PLA_1_ and the bovine brain PLA_1_ are one and the same enzyme.

Further studies eventually led to the cloning of bovine testis PA-PLA_1_, which was encoded by an 875 amino acid protein with a predicted molecular mass of 97.6 kDa [52]. A ~2000 fold increase in PA-PLA_1_ activity was observed when the protein was expressed in COS1 cells. A lipase-like consensus sequence was identified and the active site serine residue within a SXSXG motif was experimentally established as being essential for PA-PLA_1_ activity in the COS1 expression system. Apart from containing a lipase-like consensus motif, the PA-PLA_1_ sequence differed from other lipases and phospholipases. Homologues were identified in the genomes of *Caenorhabditis elegans*, yeast, *Drosophila*, and in human and mouse. A homologue involved in shoot gravitropism has also been identified in *A. thaliana* [63]. Splice variants were proposed to possibly exist after three cDNAs were found that contained a 123-base deletion, and it was put forth that if splice variants had different substrate specificities, then the seeming discrepancy between the bovine brain and testis substrate specificities may be explained in this manner. A complex array of conditions were created to examine properties of purified PA-PLA_1_ [75] using an interface composed of unilamellar lipid mixed micelles [76]. In the end, catalytic preference for PA(16:0/18:1) was reported to be observed when the unilamellar micelles contained (i) a low relative amount of PC; (ii) high relative amounts of PE, PS, and cholesterol; (iii) DAG; (iv) PE(18:0/20:4) instead of PE(16:0/18:1); and (v) 10 mol % PA per 100 mol % total phosphoglycerides. The study also showed that PA-PLA_1_ could bind to membranes composed of anionic phosphoglycerides and could be stabilized by these membranes in the presence of albumin.

Other experiments with PA-PLA_1_ provided information about the effects of phosphorylation and dephosphorylation on the behavior of the enzyme [77]. The first splice variant, PA-PLA_1_α, was recombinantly expressed in Sf9 cells and purified, though a homogeneous fraction of enzyme was not shown. The recombinant protein was shown by mass spectrometry to be phosphorylated by protein kinase CK2, with which it also formed complexes, and by extracellular signal-regulated kinase 2. They also showed that protein phosphatase 2A could dephosphorylate some of the phosphoryl modifications, which led them to raise the possibility that the native counterparts of these enzymes could coordinately regulate the phosphorylation state of PA-PLA_1_ *in vivo*. The physiological significance of PA-PLA_1_ still remains to debatably [50], because it is most highly expressed in testis it has been suggested that PA-PLA_1_ could be involved in PA signaling during spermiogenesis [74,76].

### 3.3. p125 and KIAA0725p

Budding vesicles mediate transport of proteins between intracellular compartments. One of the components of ER to Golgi COPII-coated vesicles of the secretory pathway is the Sec23p-Sec24p complex. In a study, that attempted to affinity isolate and identify new Sec23p-interacting proteins a novel protein, p125 (111 kDa), was identified. The predicted peptide sequence of p125 was homologous to bovine PA-PLA_1_ [54]. In addition, the N-terminal region of p125 contains a proline-rich region that was shown to be the functional region of the enzyme in its association with Sec23p. Detection of low levels of recombinant p125 expression showed co-localization with β-COP and ERGIC53, an ER-Golgi intermediate compartment marker. In contrast, detection of high levels of p125 over-expression revealed p125 dispersed throughout the cells, not only in membranes, but also in the cytosol, causing disorganization of the ER-Golgi intermediate compartments. ERGIC-53 also showed a dispersed staining pattern in these cells, and a second type of staining showed that the Golgi itself was dispersed. p125 was also shown to co-localize with p115 and GM130 proteins [78], both of which play a role in vesicle tethering to Golgi membranes [79]. Depletion of p125 by RNAi suggested that p125 is needed for the proper organization and distribution of ER exit sites; however, p125-depleted cells maintained regular rates of protein transport from the ER [80]. Despite these detailed studies, no examination of stereo-specific enzyme activity was undertaken, so p125 may indeed not even be a PLA_1_.

However, another protein, KIAA0725p, has also been identified as a homologue of both bovine PA-PLA_1_ and p125 [55]. KIAA0725p is ubiquitously expressed in mammalian cells and is predominantly localized in the cytoplasm. Consistent with its bovine PA-PLA_1_ homologue, KIAA0725p preferentially cleaves the *sn*-1 ester linkage of PA; however, in the absence of detergent it also hydrolyzed PE. PA specificity was determined using homogenates of KIAA0725p-expressing cells, and homogenates with the GXSXG seryl residue mutated to alanine showed no PLA_1_ activity. Like p125, results showed that over-expression of KIAA0725p in cultured cells provoked dispersion of ERGIC53 and β-COP; but, unlike p125, over-expression also caused dispersion of p115 and GM130. This morphological phenotype was not due to PLA_1_ activity because over-expression of the lipase-inactive mutant caused the same phenotype. However, over-expression of KIAA0725p, but not its lipase-inactive mutant, also caused aggregation of the ER, which was thus determined to be dependent on PLA_1_ activity. The authors proposed that KIAA0725p may promote fusion of ER membranes by changing cone-shaped PA to inverted-cone shaped lyso-PA, which has been suggested to promote fusion pore formation, the last step of membrane fusion [81]. Recently it has been shown that the isoform iPLA(1) g is a novel membrane transport factor that mediates a membrane transport pathway between the ER and the Golgi that is distinct from the previously characterized COPI- and Rab6-dependent pathways [82].

### 3.4. PS-PLA_1_

A novel PLA_1_ secreted from rat platelets was the first PLA_1_ discovered which preferentially cleaves FAs from PS [46]. The predicted peptide sequence from the cloned cDNA of PS-PLA_1_ was found to show significant sequence homology to hepatic lipase, lipoprotein lipase, pancreatic lipase, and endothelial lipase, but the enzyme does not possess appreciable neutral lipase activity. The enzyme was partially purified by sequential column chromatography from the culture medium of thrombin-activated platelets, and from medium of insect Sf9 cells expressing recombinant PS-PLA_1_ in a baculovirus system. The resulting enzyme was shown to be a 50–55 kDa protein that was equally active towards PS and lyso-PS. PS-PLA_1_ is also believed to be a glycoprotein. Due to its sequence similarity with lipases, the primary PS-PLA_1_ peptide sequence reveals that is has a β-9 loop and a lid domain; but PS-PLA_1_ has shorter versions of each [46], which could explain why diisopropylphosphofluoridate (DFP) inhibits this enzyme, if the truncated lid domain ineffectively covers the active site serine. A subsequent study revealed that PS-PLA_1_ might be the synthetic route in the production of bioactive lyso-PS since it was shown that it has the ability to efficiently stimulate histamine release from rat peritoneal mast cells, especially when in the presence of apoptotic Jurkat cells [83]. In this system, 2-acyl-1-lysoPS was released from apoptotic cells exposed to PS-PLA_1_ and it was proposed that PS-PLA_1_ may play an *in vivo* role in hydrolyzing PS exposed on plasma membranes of apoptotic, dead, and cytokine-stimulated cells, as a means to transduce mast cell activation mediated by 2-acyl-1-lysoPS. PS-PLA_1_ΔC, a splice variant of the human version of PS-PLA_1_, shows poor catalytic activity towards PS and the other diacylPLs but is able to deacylate lyso-PS effectively and is therefore a LysoPLA [84]. PS-PLA_1_ΔC possesses a truncated C-terminal region; therefore peptides on the complete C-terminal domain of PS-PLA_1_ are thought to play a role in substrate recognition. In a very recent study PS-PLA_1_ expression in human THP-1-derived macrophages, responsible for the immune responses to allograft rejection, are activated via TLR4. This activation can be inhibited by corticosteroids, which are used at high dosages to suppress chronic allograft rejection [57].

### 3.5. mPA-PLA_1_

A sequence similarity search of the PS-PLA_1_ detected another PLA_1_ enzyme which was homologous to neutral lipases, that of membrane-associated phosphatidic acid-selective phospholipase A_1_ alpha and beta (mPA-PLA_1_α/β) [43,56]. Both recombinant forms show PA-specific substrate specificity. Attempts to purify the recombinant membrane proteins from Sf9 cells failed but medium from mPA-PLA_1_α-expressing cells was shown to activate a lyso-PA receptor family member, LPA3/EDG7. Medium from cells expressing an active site serine mutant failed to induce a receptor response. These results indicated that cells expressing recombinant mPA-PLA_1_α were able to produce and release bioactive lyso-PA. When expressed in HeLa cells mPA-PLA_1_α was recovered from the cell supernatant whereas mPA-PLA_1_β was still membrane associated [56]. A set of elegant experiments also showed that mPA-PLA_1_α/β acted after and in concert with both endogenous and exogenous PLD to form lyso-PA. This report thus suggests a possible *in vivo* metabolic pathway for lyso-PA production involving the sequential action of a PLD and mPA-PLA_1_α/β. Thus, both PS-PLA_1_ and mPA-PLA_1_α/β thus have evolved from lipases to specialize in producing bioactive lyso-PL mediators [5].

### 3.6. Guinea-Pig Heart Microsomal PLA_1_

It has been reported that microsomes from guinea-pig heart possess PLA_1_ activity on PE and PC, and that *sn*-1 cleavage could be influenced by the *sn*-2 FA since more efficient hydrolysis was observed when the *sn*-2 FA was polyunsaturated [85]. The enzyme(s) responsible for PLA_1_ activity from guinea-pig heart microsomes have not been identified and purified to any extent other than into subcellular fractions, but a few studies are worth mentioning since they are devoted to possible regulatory mechanisms of PLA_1_ activity in this tissue, and very few other studies exist that begin to explain how some PLA_1_ activity could be controlled. No PLA_1_-activating agonists are known, so a possibility that PLA_1_ could be receptor activated by G-proteins was investigated by measuring the response of PLA_1_ activity towards guanine nucleotides, which can activate G-proteins. PLA_1_ activity towards PC(16:0/18:2) was partially inhibited by guanosine 5′-[γ-thio]triphosphate (GTP[S]), but not by GTP, guanosine 5′-[γ-thio]diphosphate (GDP[S]), GDP, ATP or adenine 5′-[γ-thio]diphosphate (ATP[S]) [86]. On the other hand, PLA_1_ activity on PE(16:0/18:2) was stimulated by GTP[S] but not GDP[S] or ATP[S] [86]. PE(16:0/18:2) hydrolysis by guinea-pig heart microsomes was also shown to be stimulated 40–60% by isoprenaline. It has still yet to be shown *in vivo* if PLA_1_ activity in guinea-pig heart microsomes is activated by the binding of isoprenaline to adrenergic receptors and mediated via activation of G-proteins. Without either the purification or cloning of the enzymes responsible for this activity in guinea-pig heart microsomes, it is unlikely that any resolution of the issue will be made. It is worth recalling, however, that a ubiquitously expressed mammalian PA-PLA_1_ homologue, p125, is localized to the ER and interacts with Sec23p, which acts as a GTPase-activating protein that plays a role in uncoating budding vesicles [87]. Whether these PLA_1_ activities are due to the same or different enzymes is unknown, but it does stress the importance of how substrate specificity is analyzed *in vitro*, and the caution with which to interpret such data. To confuse the situation even further, guinea-pig heart microsomes were also shown to possess a LysoPLA_2_ that could hydrolyze lyso-PC(−/18:2 or 20:4) more efficiently than lyso-PC(−/18:1 or 16:0) [18].This result lent some support to the standing possibility that arachidonic and linoleic acids could be released by LysoPLA_2_ acting in concert with a PLA_1_.

## 4. Other PLA_1_

PLA_1_ is a ubiquitous enzyme found in nearly every cell where it has been sought, and this includes metazoan and protozoan parasites, and snake venoms.

### 4.1. *Caenorhabditis elegans* PLA_1_

The *Caenorhabditis elegans*, IPLA-1 and ACL-10 have phospholipase A_1_ and acyltransferase activity respectively, both of which recognize the *sn-*1 position of their PI substrate [88]. The PI *sn*-1 fatty acid remodeling by sequential deacylation and reacylation, which resulted in stearic acid as the major fatty acid at the *sn*-1 position, has been proposed to be crucial for asymmetric division [5].

### 4.2. Venom PLA_1_

The cDNA of one allergen component of *Dolichovespula maculate* (white-faced hornet) venom has been cloned and shown to encode a PLA_1_ (Dol m I) with weak lipase activity [47]. In fact, at the time of the study, the derived peptide sequence of Dol m I had no homology to other PLA_1_ genes because it was similar to mammalian lipases. Now, not only is Dol m I 40% identical to pancreatic, hepatic, lipoprotein, and endothelial lipase, but it also shows homology with PS-PLA_1_ [46] and mPA-PLA_1_ [43]. Characterization of the 34–37 kDa enzyme has not been undertaken presumably due to a priority interest in understanding its immunochemical properties as it relates to its contribution to allergenicity. Along these same lines, two homologues of Dol m I have been cloned from *Vespula* spp. (yellow jacket) (Ves v I and Ves m I) which show 67% sequence identity with the white-faced hornet PLA_1_ [58,59], and recent success in recombinant expression of PLA_1_ (Ves v I) from the yellow jacket will allow a more detailed understanding of the molecular and allergological mechanisms of insect venoms, providing a valuable tool for diagnostic and therapeutic approaches in hymenoptera venom allergy [60].

### 4.3. *Trypanosoma brucei* PLA_1_

The PLA_1_/LysoPLA theorized coordinated route towards the release of unsaturated FAs, (e.g., arachidonic acid (AA)), has been supported by *in vitro* studies using homogenates from the kinetoplastid protozoan parasite *Trypanosoma brucei*, the infective agent of African sleeping sickness [23]. These studies observed robust and optimal activity at pH 6.0–8.5 and a requirement of *sn*-1 acyl cleavage prior to the release of AA from *sn*-1-palmitoyl-*sn*-2-arachidonoyl-*sn*-glycero-3-phosphatidylcholine [PC (16:0/20:4)] [89–91]. The liberated unsaturated FAs such as AA from lipids in *T. brucei* have been implicated in regulating calcium mobilization [92,93], and as a precursor for prostaglandin biosynthesis [94].

Two recent detailed studies describe the cloning and characterization of the cytosolic *T. brucei* PLA_1_. TbPLA_1_ is unique from other eukaryotic PLA_1_ because it is phylogenetically related to bacterial secreted PLA_1_ [49,66]. *TbPLA_1_* was most likely acquired by a prokaryotic-to-eukaryotic horizontal gene transfer event of a *PLA_1_* from *Sodalis glossinidius*, a bacterial endosymbiont of the insect vector of the protozoan parasite, the tsetse fly. These studies employed both *in vitro* and *in vivo* analytical techniques to establish that PC is the preferred substrate. The *TbPLA_1_* homozygous null mutants constitute the only *PLA_1_* double knockouts from any organism and helped to established that the enzyme functions *in vivo* to synthesize lyso-PC metabolites containing long-chain mostly polyunsaturated and highly unsaturated fatty acids.

### 4.4. *Trypanosoma cruzi* PLA_1_

One report described the existence of PLA_1_ activity in *Trypanosoma cruzi*, the etiologic agent of Chagas disease [95]. When trypomastigote and amastigote suspensions were radiolabeled with oleic acid and lysed, the radiolabeled PC content decreased over time. In addition, when PC(16:0/18:1) was incubated with epimastigote homogenates at pH 4.7, lyso-PC(−/18:1) was produced, suggesting *sn*-1 hydrolytic specificity. *T. cruzi* PLA_1_ was partially purified (1900-fold by specific activity) from epimastigote homogenate supernatants and a protein band of 38 kDa could be seen by SDS-PAGE, and size exclusion chromatography suggested PLA_1_ activity eluted with an apparent molecular mass of 40 kDa. *T. cruzi* PLA_1_ seems to be a glycoprotein due to its binding to ConA-Sepharose, possesses no divalent cation requirements, and is released from the cell by digitonin together with lysosomal markers. The physiological role played by lysosomal *T. cruzi* PLA_1_ has not been determined, though the authors suggest that the FA and lyso-PL products could have a possible role in pathogenesis of the disease.

## 5. Plant Phospholipase A_1_

Evidence for the existence of PLA_1_ in plant cells wasn’t forthcoming until relatively recently when the tonoplasts of *Acer pseudoplatanus* were shown to be able to hydrolyze *sn*-2-radiolabeled PC into radiolabeled PA and radiolabeled lyso-PC, revealing PLD and PLA_1_ activities, respectively [96]. Since then, only a few genes encoding PLA_1_ have been discovered.

### 5.1. DAD1

Among the plant hormones jasmonic acid is considered a multifunctional growth and stress regulator [97], and it is structurally similar to “animal” eicosanoids. Jasmonic acid is an oxylipin signaling molecule and a derivative of linolenic acid (C18:3). Jasmonate has been shown to regulate or co-regulate a variety of processes in plants, such as responses to biotic and abiotic stresses, tendril coiling, fruit ripening, and the developmental maturation of stamens and pollen in *Arabidopsis*. The enzymes involved in the jasmonic acid biosynthetic pathway have been elucidated, but the last one discovered, a phospholipase A_1_ called Defective in Anther Dehiscence 1 (DAD1), was one of the most important because it is the enzyme responsible for the initial release of C18:3 from cellular lipids [61]. Starting from an *Arabidopsis thaliana* DAD1 mutant, the WT DAD1 gene was isolated and found to encode a chloroplastic 45 kDa PLA_1_ lipolytic enzyme. A rescued phenotype was attained by either complementing the mutant with WT DAD1 or after supplying exogenous jasmonic acid. The DAD1 protein sequence showed some apparent similarities with fungal lipases, it possessed a consensus GHSLG motif, and eleven homologous proteins were identified in *Arabidopsis* alone. A maltose binding-DAD1 fusion protein lacking 72 amino acid residues (an N-terminal transit peptide) was recombinantly expressed and used to examine lipase activity. PC was the only PL tested, and the activity on it was more than 80% greater than on TAG, which suggested that the C18:3 precursor for jasmonic acid biosynthesis is stored in cellular PLs. DAD1 is thought to be of prime importance for the regulation of jasmonic acid levels. A PLA_1_ from *Capsicum annuum* (hot pepper) showed a considerable degree of overall sequence identity to Arabidopsis [98].

### 5.2. AtLCAT3

In a search for *A. thaliana* sterol acyltransferases, one of the four enzymes found to be homologous to lecithin (PC): Cholesterol acyltransferases was determined to possess PLA_1_ activity [62,63]. AtLCAT3 (*Arabidopsis thaliana* lecithin:cholesterol acyltransferase 3) heterologous expression in yeast resulted in the PC, PS, and PE content to be half as much as those species in control yeast, while lyso-PC, lyso-PE and FFA were strongly increased. There was also a higher TAG content in the cells expressing AtLCAT3. AtLCAT3 fractionated with yeast microsomes, which subsequently shown to be able to hydrolyze various PL species, including lyso-PC and PA. However, neither acyltransferase activity nor TAG activity was observed with yeast microsomes expressing the enzyme. The analogous serine, histidine and aspartic acid residues that are part of the conserved catalytic triad of *Homo sapiens* LCAT were shown to be essential for activity of AtLCAT3. The physiological role of AtLCAT3 is as yet unknown.

## 6. Bacterial PLA_1_

The discovery of prokaryotic versions of PLA_1_ enzymes have up to now been confined to only one subclass of bacteria, the proteobacteria [68]. PLA_1_ from *Serratia liquefaciens* (PhlA) was identified as one of a number of proteins that are excreted to the outside environment. The impetus for studying PhlA was to use *S. liquefaciens* as a model organism to study the genetic basis for secreting proteins across both the periplasmic and outer membranes in gram-negative bacteria. As a result, very little biochemical information regarding PhlA is known. In fact, phospholipase activities of this enzyme were only examined against egg yolk PC imbedded in agar plates in which transparent halos were observed after the addition of cells expressing the enzymes. The presence of a halo around the colony indicated secreted phospholipase A activity, but did not discern between *sn*-1 and *sn*-2 specificity. *PhlA* encodes a 34 kDa polypeptide that has an N-terminal signal peptide. Immediately downstream from the *PhlA* gene is *PhlB*, which encodes a protein of 24 kDa.

The bacterial exoenzyme PhlA was shown to have growth-phase-dependent expression and secretion, where a very low rate of PhlA production was measured during exponential growth, and a burst of expression and secretion was observed during stationary phase. In another report it was shown that PhlA expression is regulated at the level of transcription initiation, and they present sequence data of two dual promoters upstream of *PhlA* that regulate expression differentially during anaerobic conditions or growth-phase [99]. A subsequent study revealed that PhlA secreted from in *E. coli* was dependent on an intact *flhD* gene, the regulator of the flagellar/chomotaxis opero [67,100]. Mutant *flhD* strains of *E. coli* do not secrete recombinant PhlA, which accumulates inside the cell. Interestingly, the PLA_1_ activity of accumulated PhlA is attenuated by PhlB, which forms an enzymatically inactive complex with PhlA, thus neutralizing possibly lethal intracellular phospholipase activity.

A homologue (74% identity) of PhlA was identified in *Yersinia enterocolitica* (YplA) [70]. The YplA sequence revealed two ORFs in tandem like that of the PhlA and PhlB, but the YplB accessory protein was less similar to PhlB. Again, the phospholipase A activity was confirmed by testing on lecithin plates only. An insertion mutant of YplA was created and used in a mouse model to show that secreted YplA could play a role as a virulence factor in the pathogenesis of infection with *Y. enterocolitica*. Neither PhlA nor YplA are homologous to bacterial lipases.

## 7. PLA_1_ in Biotechnology

LysoPLs are commonly used as surfactants in food technology and cosmetics [101], and as components of liposomes used in drug delivery [102]. Lyso-PLs are currently produced commercially via their chemical synthesis [103] or via the action of extracts from porcine pancreas, which contains a PLA_2_. Viable alternative processes produce lyso-PLs enzymatically and with greater efficiency are being investigated, recently reviewed in [104].

Phospholipases are useful tools in analytical PL analysis. Though there are a number of sources of PLA_2_ commercially available at various levels of purification, no PLA_1_ in any form is commercially available, mainly due to the difficulty in producing and purifying the enzyme, especially on an industrial scale. The ciliated protozoan *Tetrahymena thermophila* has been studied as a potential natural source for PLA_1_ [105], which is secreted from this organism. However, high levels of PLA_1_ are undoubtedly best obtained through recombinant expression techniques. Since purified PLA_1_ is expected to have broad industrial applications, a number of PLA_1_ genes from a variety of microorganisms have been cloned and attempted to be expressed and purified to fulfill this need, but they have not been characterized.

*Serratia* spp. MK1 PLA_1_ (PlaA), a homologue of PhlA and YplA, has been isolated from *Serratia* spp. MK1 in Korean soil and cloned and expressed in *E. coli* [69]. Though the enzyme is secreted naturally, over-expression resulted in accumulation of recombinant enzyme inside the cell, and co-expression of its accessory protein, PlaS, was essential for cell viability and high expression levels. The histidine affinity tag on the recombinant protein was used in a one-step purification of the enzyme, which yielded under the best conditions 2.2 mg/L, the purity level was neither reported nor shown. Also, PLA_1_ in organisms adapted to lower temperatures have recently been found [106].

The genomic DNA and cDNA encoding a 269 amino acid PLA_1_ protein from *Aspergillus oryzae* have also been cloned [64]. The peptide sequence of the PLA_1_ showed 47% identity with that of mono- and diacylglycerol lipase from another fungus, *Penicillium camembertii. A. oryzae* PLA_1_ is a secretary enzyme and it was recombinantly expressed in the yeast *Saccharomyces cerevisiae* and purified from the extracellular broth at a level of 3.9 mg/L. Under a complex array of conditions, this expression was optimized to produce approximately 2 g/L on an industrial scale fermentor [107]. However, the enzyme was only partially purified from the culture broth by ion exchange chromatography and the yield was calculated based on its specific activity. Recently a PLA_1_ from *Thermomyces lanuginosus* expressed in *Aspergillus* has been commercialized as a lectinase [65].

## 8. Perspective

Phospholipases A_1_ are enzymes that hydrolyze phospholipids at the *sn*-1 fatty acids from phospholipids and produces 2-acyl-lysophospholipids. This PLA_1_ activity is conserved in a wide range of organisms, but is carried out by a diverse range of enzymes.

Despite PLA_1_ activities being detected in many tissues and cell lines, only a limited number of PLAs have been cloned and their activity and substrates identified and characterized, and even fewer have been studied that their function is known.

PLA_1_s have been shown to have a diverse range of cellular functions including, digestive enzymes, central roles in membrane maintenance and remodeling via the Lands cycle of important phospholipids or glycolipids such as glycosylphosphatidylinositols. It is now emerging that they also regulate and facilitate the production of various lysophospholipid mediators, such as lysophosphatidylserine and lysophosphatidic acid, which in turn have multiple important biological functions.

Thus, phospholipases A_1_ are an emerging class of enzyme that play important roles in the cellular functions including those of various diseases and pathogens that affect human health.

## Figures and Tables

**Table 1:** The Phospholipase A_1_ Family 1.

Classification	Organism	Name 2	Location	Cellular Localization	~ Size (kDa)	Substrate Specificity	Catalytic Properties	Reference
**Eukarya**								
Animali:	*Bos taurus*	□ PA-PLA_1_	Testis, Brain	Cytosolic	98	PA	SXSXG catalytic serine^3^	[52,53]
	*Mus musculus*	□ p125	Ubiquitous	Cytosolic	111	nd^4^	GXSXG motif	[54]
	*Homo sapiens*	□ KIAA0725p	Ubiquitous	Cytosolic	81	PA, PE	GXSXG catalytic serine^3^	[55]
	*Homo sapiens*	• mPA-PLA_1_α	Various tissues	Secreted	58	PA	Catalytic triad^5^	[43,56]
		• mPA-PLA_1_β	Reproductive tissues	Mem-Ass	58	PA	Catalytic triad^5^	[43,56]
		PLRP2						[50]
	*Rattus norvegicus*	• PS-PLA_1_	Platelets+various	Secreted	55	PS, lysoPS	Catalytic triad^5^	[46,48,57]
	*Dolichovespula maculate*	• Dol m I	Venom sac	Secreted	34	nd^4^	Catalytic triad^5^	[47]
	*Vespula* spp.	• Ves v I	Venom sac	Secreted	34	nd^4^	Catalytic triad 5	[58–60]
		• Ves m I	Venom sac	Secreted	34	nd^4^	Catalytic triad 5	[58–60]
	*Caenorhabditis Elegans*	IPLA-1		ER	87	PI	Catalytic triad 5	[5]
Plantae:	*Arabidopsis thaliana*	DAD1	Anthers	Chloroplast	45	PC^6^	GXSXG motif	[61]
		AtLCAT3	nd^4^	Microsomes	46	PC, PE, PA	SXSXG-catalytic triad^3^	[62,63]
Fungi:	*Aspergillus oryzae*	AoPLA_1_	n/a^4^	Secreted	36	nd^4^	GXSXG motif	[64]
	*Thermomyces lanuginosus*		n/a^4^	nd^4^		PC		[65]
Protozoa:	*Trypanosoma brucei*	TbPLA_1_	n/a^4^	Cytosolic	34	PC	GXSXG catalytic serine 3	[49,66]
**Prokarya**								
Bacteria:	*Serratia* spp.	♦ PhlA	n/a	Secreted	34	nd^4^	GXSXG motif	[67,68]
		♦ PlaA	n/a	Secreted	34	nd^4^	GXSXG motif	[69]
	*Yersinia enterocolitica*	♦ YplA	n/a	Secreted	34	nd^4^	GXSXG motif	[70]

1 Only those PLA_1_ which have been cloned and reported in the literature have been included;

2 Homologues are represented with the same symbol. Abbreviations are: PA-PLA_1_,phosphatidic acid-preferring PLA_1_; mPA-PLA_1_, membrane-associated phosphatidic acid-selective PLA_1_; PS-PLA_1_; phosphatidylserine-specific PLA_1_; DAD1, defective in anther dehiscence 1; AtLCAT3, *Arabidopsis thaliana* lecithin:cholesterol acyltransferases; SGR2, shoot gravitropism 2; PhlA, *Serratia liquefaciens* PLA_1_; PlaA, *Serratia* spp. MK1 PLA_1_; YplA, *Yersinia enterocolitica*;

3 Empirically deduced;

4 nd = not determined, n/a = not applicable;

5 Deduced by similarity with its lipase homologues known to utilize a histidine, an aspartic Acid, and a GXSXG serine in a catalytic triad;

6 The only substrate tested.
